# Outside enclosure and additional enrichment for dairy goats – a preliminary study

**DOI:** 10.1186/1751-0147-54-68

**Published:** 2012-11-22

**Authors:** Knut Egil Bøe, Rebecca Ehrlenbruch, Inger Lise Andersen

**Affiliations:** 1 Department of Animal- and Aquacultural Sciences, Norwegian University of Life Sciences, P.O. Box 5003, 1432, Ås, Norway

**Keywords:** Goat, Outside enclosure, Environmental enrichment

## Abstract

**Background:**

Dairy goats are commonly housed at a space allowance of 0.7 – 0.8 m^2^/goat in commercial Norwegian goat herds, which is very low compared to regulations and recommendations in other European countries. One easy and cheap way to increase space allowance is to allow the animals’ access to outdoor area. The aim of this study was to investigate the effect of access to an outside enclosure and environmental enrichment for dairy goats kept in slatted floor pens with low space allowance on their activity pattern and social behaviour.

**Methods:**

A group of 82 dairy goats on a commercial Norwegian dairy farm were kept inside during the winter period from October to April. In April the goats were given access to an outside enclosure for 8 hours per day. After having access to the enclosure for another for two days, enrichment (branches) was provided, and after 19 days the enrichment were removed. The goats were observed for 5 hours per day for the two last days before they got access to the outside enclosure, the two days in the enclosure, the two first and the two last days with enrichment and for the following two days without enrichment by two trained observers.

**Results:**

When allowed access to the enclosure, the goats spent nearly 50% of the time outside, and later the time spent outside was reduced to less than 40% (P < 0.0001), but there was no clear effect of enrichment. All the goats appeared to have a regular use of the enclosure. Time spent resting decreased 59.2% to only 25.2% when the goats first got access to the enclosure, but then started to increase again (P < 0.0001). Initially time spent exploring and chewing the branches was 20%, but this was reduced to around 12% in the last part of the ENRICH period (P < 0.0001). Number of aggressive interactions tended to increase when the goats were allowed access to the outdoor enclosure whereas play behaviour was only observed in the outside enclosure (P < 0.05).

**Conclusions:**

In conclusion, the goats preferred to use the outside enclosure when being active, and branches were perceived as an attractive enrichment.

## Background

In a survey of Norwegian goat herds, Simensen et al. (2010) found that the vast majority (94%) of the herds kept their goats in insulated buildings during winter time with no access to an outdoor area [1]. Even if no data on space allowance is provided in this survey, goats are commonly housed at a space allowance of 0.7 – 0.8 m^2^/goat. New regulations for organic goat farming (Council Regulation (EC) No. 1804/1999) demand a minimum of 1.5 m^2^ total area per animal, and half of this should be a resting area with a solid floor (0.75 m^2^ per goat). This is in accordance with French recommendations [2]. This demand for space is supported by studies made by Andersen and Bøe (2007) who found that when the resting area was less than 1.0 m^2^ per goat, lying simultaneously decreased and lying in the activity area increased [3]. Further, Loretz et al. (2004) reported that lying time was reduced when the size of the lying area was reduced from 2.0 m^2^/goat to 1.0 m^2^/goat [4]. One easy and cheap way to increase space allowance is to allow the animals’ access to outdoor area. In dairy cows several papers can document that access to an outdoor yard is beneficial for the health and welfare of the cows e.g. [5-7]. Use of outdoor yards during winter time is not common in Norway, probably because of large amounts of snow and low temperatures during winter, but in Switzerland the use of outdoor yards are considered to be good farming practice and supported by the authorities (RAUS-Programm, Ethoprogrammverordnung). Also turnout for horses is regarded positive. Studies of sheep [8] with access to outdoor yards during winter time showed that ewes spent a considerable amount of time in the outside yard irrespectively of the weather conditions.

During the winter season Norwegian goat herds mainly keep their goats in fully slatted for pens [1] mainly to ensure good hygienic conditions, but also because of lack of appropriate bedding material. Type of flooring do not seem to have a large impact on the goats resting behaviour under temperate climatic conditions [9], but fully slatted floor pens must definitely be regarded as a barren environment. Hence, access to an outdoor yard will not only provide more space for exercise but also represent an enriched environment. Observations of horses in outdoor paddocks [10] showed that their activity level was low unless enrichment items were provided. Access to an outdoor enclosure will provide more space, which is shown to increase resting time and time spent lying simultaneously for goats [3,4]. Outdoor enclosures represents a more stimulating, heterogeneous environment, especially as goats spend a considerable proportion of their time browsing e.g. [11] and browsing woody species is an important forage source for goats (for review see [12]).

The aim of this study was to investigate the effect of access to an outside enclosure and environmental enrichment for dairy goats kept in slatted floor pens with low space allowance on their activity pattern and social behaviour.

## Methods

### Animals, feeding and management

This study was performed on a commercial dairy goat farm, localized in Folldal in the middle of Norway (latitude: 62°07^′^ N, longitude: 09°59^′^ E) 700 m above sea level. A total of 82 lactating goats of the Norwegian dairy breed, 1 – 2 years of age, both horned and hornless, were kept together in one group. Prior to the experiment the goats had been kept inside from beginning of October (end of grazing season) to the start of this experiment in April. Normally the goats were let out on pasture from beginning of June to the end of September.

Good quality hay and silage were offered *ad libitum* on the feeding table running along the pen inside the barn. In addition, the goats were fed a standard concentrate feed (1.4 kg per goat daily) four times (06.00 h, 12.00 h, 17.00 h and 22.00 h) per day. The goats had free access to water from six nipple drinkers installed in the pen. Twice a day the goats were milked (06.30 - 07.00 h and 17.30 – 18.00 h) in a milking parlour located in an adjacent section.

### Experimental pen and enclosure

**Figure 1:**
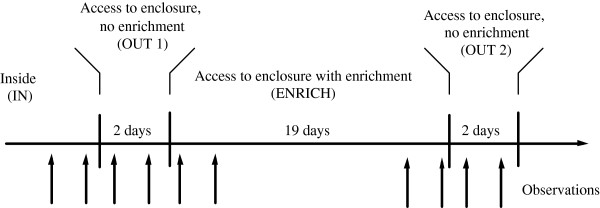
Experimental design and observation days.

The goats were kept in an insulated, mechanically ventilated building with an ambient air temperature around + 9°C, and all the lactating goats (82 animals) were kept in a 76.4 m^2^ (0.94 m^2^ per goat) pen with slatted plastic flooring. Through a door (1.0 m × 1.9 m) the goats had access to an outside enclosure of approximately 750.0 m^2^ (around 9.0 m^2^ per animal). In the enclosure there was some snow that were building up a small hill, small rocks and some grass (Figure 1). Branches of pine and/or birch (4–5 pieces approx. 2.0 m long) intact with pine needles and bark were offered daily each morning at 09.00 h in ENRICH treatment. 

### Experimental design

**Figure 2:**
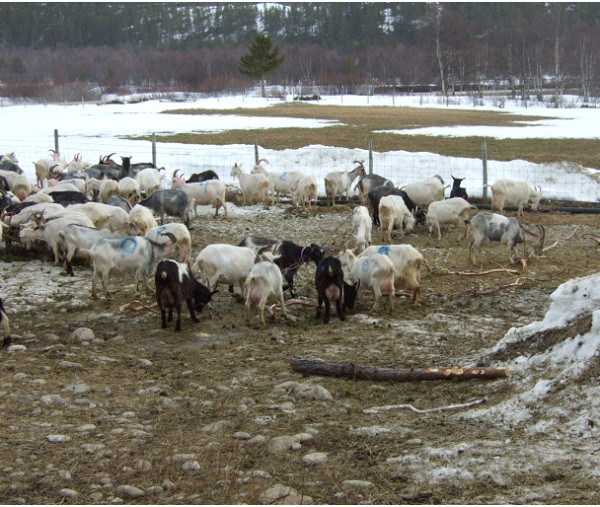
The goats in the outside enclosure.

Dairy goats were kept inside an insulated building during the winter period from October to April as is standard management in Norway (IN - inside). In the beginning of April the goats were given access to an outside enclosure for two days (OUT1 – access to enclosure, no enrichment) (see Figure 2). Thereafter the goats continued to have access to the enclosure for another 19 days, but now enrichment (branches) was provided (ENRICH – access to enclosure, with enrichment). In treatment OUT2 (access to enclosure, no enrichment) the goats continued to have access to the enclosure for two days, but now the enrichment was removed. Providing enrichment for nearly three weeks was done in order to investigate the long term effect of enrichment. 

In the treatments with access to enclosure, the door to the enclosure was open daily for 8 hours, from 09.00 h (after the morning feeding and milking) to 17.00 h in the afternoon (before afternoon feeding and milking).

### Behavioural observations

Twenty goats of the group of 82 were randomly selected (10 horned and 10 hornless) for individual observations, and marked across their back with a marker spray for animals and dark goats were marked with different coloured collars. The goats were observed the two last days before they got access to the outside enclosure (IN), both days in OUT1, the two first (ENRICH1) and the two last days (ENRICH2) of the period with access to the enclosure and with enrichment and both days in OUT2 (ten days in total, see Figure 1). On all the observation days it was cloudy or partly cloudy and no precipitation was recorded. The outside air temperature varied from 0°C to 8°C and there was little wind (mean: 0.8 m/sec).

Two trained observers, standing inside the barn with a good view of the enclosure from the windows, scored the location (inside/outside) and the behaviour of the marked goats every 10^th^ minutes (instantaneous sampling) for 5 hours (09.00 - 11.30 and 12.30 - 15.00 h) each observation day using the following ethogram with mutually exclusive behaviours:

– Resting

– Standing/Walking

– Eating roughage (head over feeding table) or drinking (only inside)

– Eating old grass (only outside)

– Social activity (aggressive interactions such as butting and clashing, and positive interactions such as social grooming and exploratory sniffing)

– Exploring environment inside (sniffing, licking chewing on or scratching with the hoof against pen fittings)

– Exploring in the enclosure (sniffing, licking, chewing on or scratching with the hoof against the fence, rocks or snow)

– Exploring or chewing branches (only outside)

Aggressive interactions (butting, clashing, pushing, biting and physical displacements from feed barrier or resting place) were scored continuously and the initiator and receiver of these aggressive interactions. Also play behaviour (jumps and bounces) were recorded continuously.

In addition, the total number of goats in the group (82 goats) that were inside or outside was scored every 10 minute.

### Statistical analysis

To analyse the effect of access to an outside enclosure, with and without enrichment, on the activity pattern and social behaviours, a mixed model analysis of variance with treatment (IN, ENRICH1, OUT1, ENRICH2 and OUT2) and goat (1–20) was used [13]. ‘Goat’ was specified as a random effect in the model. Mean values per goat within treatment were used as statistical unit.

## Results

### General activity

When allowed access to the enclosure (OUT 1 and ENRICH1), the goats spent nearly 50% of the time outside, but later (ENRICH2 and OUT2) the time spent outside was reduced to less than 40% (Table 1). There was no clear effect of enrichment on time spent outside. All the goats appeared to have a regular use of the enclosure and all goats were observed to be outside every observation day. The goat that used the enclosure most was outside 60.8% of the observations (mean of all observation days) whereas the goat that used the enclosure least spent 24.2% of the observation time outside (mean of all observation days). 

**Table 1:** The overall resting and activity pattern (mean ± SE in % of total observations)

	**IN**	**OUT1**	**ENRICH1**	**ENRICH2**	**OUT2**	**F**	**P**
In enclosure		49.1 ± 12.7^a^	47.2 ± 9.2^a^	37.1 ± 11.2^b^	38.4 ± 12.8^b^	10.70	<0.0001
Resting	59.2 ± 10.8^a^	25.2 ± 10.0^e^	30.7 ± 8.4^d^	36.0 ± 6.5^c^	43.8 ± 8.1^b^	74.87	<0.0001
Standing/Walking	22.0 ± 2.3^a^	51.0 ± 2.3^c^	33.3 ± 1.6^b^	31.2 ± 1.5^b^	32.0 ± 25.0 ^b^	42.87	<0.0001
Eating roughage or drinking (inside)	12.5 ± 1.1^a^	12.3 ± 1.2^a^	10.0 ± 1.1^a^	13.5 ± 1.2^b^	14.0 ± 0.9^b^	2.63	< 0.05
Eating grass (in enclosure)	-	1.8 ± 0.4^a^	0.9 ± 0.2^b^	0.0 ± 0.0^c^	0.7 ± 0.2^b^	9.71	<0.0001
Exploring environment inside	0.3 ± 0.2	0.1 ± 0.1	0.1 ± 0.1	0.2 ± 0.1	0.2 ± 0.1	0.63	ns
Exploring environment in enclosure	-	6.4 ± 0.9^a^	2.2 ± 0.3^bc^	1.2 ± 0.3^c^	3.3 ± 0.7^b^	15.41	<0.0001
Exploring or chewing branches	-	-	20.3 ± 6.3	12.7 ± 6.5	-	32.54	<0.0001
Social activity	5.9 ± 1.4^a^	3.3 ± 0.7^b^	2.5 ± 0.6^b^	5.3 ± 0.8^a^	6.0 ± 1.0^a^	4.05	<0.01
Aggressive interactions (number per goat and 5 hour)	11.2 ± 1.8	27.7 ± 8.4	23.8 ± 5.8	23.8 ± 4.3	34.8 ± 6.6	2.44	<0.10
Playing (number per goat and 5 hour)	0.0 ± 0.0^b^	0.7 ± 0.2^a^	0.9 ± 0.3^a^	1.1 ± 0.4^a^	0.8 ± 0.3^a^	3.43	<0.05

Based on the observations of the whole group (82 goats), the mean proportion of goats being in the outside enclosure was 41.3%, 37.0%, 29.8% and 33.3% in the OUT1, ENRICH1, ENRICH2 and OUT2 period respectively. All goats were rarely observed simultaneously in the enclosure (mean 1.7, range 0 – 4.9% of observations).

Time spent resting decreased 59.2% to only 25.2% when the goats first got access to the enclosure, but then started to increase again (Table 1). The goats were mainly resting inside. 16 of the 20 goats were resting in the enclosure at least during one observation, but generally lying in the enclosure was rare (mean: 3.0% of observations). Time spent eating roughage was not affected by treatment (Table 1).

Proportion of time spent standing/walking increased to more than 50% when the goats got access to the outdoor enclosure (OUT1) and later it leveled out to around 30% of the observations (Table 1). The goats spent little time exploring the environment.

When the branches initially was introduced in the enclosure (ENRICH1), the goats spent around 20% of the time exploring and chewing the branches, but this was later (ENRICH2) reduced to around 12% (Table 1). All the goats took part in this activity. In the first period (ENRICH1) up to 19 goats were observed exploring and chewing the branches simultaneously (mean 4.1 goats) whereas in period 2 (ENRICH2) the maximum number was 12 (mean 2.5 goats). The goats spent more time exploring the environment when stimuli (the branches) were not present in the enclosure (Table 1). Further, the goats spent a very limited amount of time eating grass in the enclosure, but there was no clear effect of treatment.

Time spent in social activity varied significantly, but there was no clear effect related to access to enclosure or provision of branches.

### Social interactions and play

Number of aggressive interactions tended to increase when the goats were allowed access to the outdoor enclosure (Table 1). Play behaviour was never observed when the goats were kept constantly inside, but was observed several times in the outside enclosure (Table 1). Seventeen of the 20 goats were observed to perform play behaviour when allowed access to the enclosure.

## Discussion

When given access to an outdoor enclosure, the goats spent a large proportion of their time in the enclosure, increased time spent active and consequently a decrease in time spent resting. This might be due to both the increased space allowance *per se* but also the enriched environment in the enclosure. Loretz et al. (2004) found that goats spent more time resting when space allowance was increased [4] while studies on other farm species dry sows: [14]; calves: [15] indicate that increased space allowance had no effect on total activity. What makes the conditions in the present study special is that the space allowance was increased considerably when allowing access to the enclosure (from 0.9 m^2^ per animal to nearly 10 m^2^ per animal), the goats had only access to the enclosure during some hours in the middle of the day, and access to the enclosure was introduced several months after the goats had been kept in a rather restricted space. The latter might also partly explain that total activity decreased in the last part of the experimental period, both with and without environmental enrichment (branches), a possible rebound effect as discussed by [16]. Interestingly, Loberg et al. (2004) found that tied cows allowed to exercise in a paddock for one hour every 7^th^ day was more active in the paddock than cows allowed access every day [5]. Data from horses e.g. [10][17] support these results. Another reason for the declining activity might be the reduced novelty of both the outside enclosure *per se* and the environmental enrichment (branches) as total activity was reduced both within the OUT-treatment and the ENRICH-treatment.

Studies of dairy calves do show that locomotor play was higher when space allowance was increased [15][18] and that there also were a rebound effect in that calves reared for longer time in small pens showed more galloping and bucking in an open-field test [16]. Also in the present study play behaviour was when the goats had access to the outdoor enclosure, although at a low frequency.

Time spent exploring the indoor environment was very low, as could be expected due to the barren environment (expanded metal flooring and no bedding). When given access to the outdoor enclosure, the goats spent some time both exploring other features of the enclosure and even eating/exploring the old grass, and their overall activity level was enhanced. Then, when enrichment was provided (branches), the goats spent a considerable amount of time exploring and chewing these branches, and time spent lying/standing was further reduced. As goats are often browsing [11], it was not surprising that they spent much time exploring the branches supplied in the enclosure. Correspondingly, horses kept in groups during turnout reduced the amount of passive behaviours (standing or lying) when exposed to edible enrichment items such as branches or a ball with concentrates [19]. However, it is important to notice that the horses, even when exposed to edible items, spent a considerable amount of time eating green leaves from the surface. Also studies in pigs [20] confirm that edible enrichment objects are well-used and reduce behavioural problems. Loberg et al. (2004) showed that tethered cattle spent 15 to 30% of the time just exploring the ground and objects when offered access to an outdoor paddock, and that time spent exploring increased when cows only were let out in the paddock once weekly compared to daily exposure [5]. Exploring and chewing branches was significantly reduced from when these items were first introduced and when the goats were observed 19 days later, which suggest a clear effect of reduced novelty of the enrichment.

Time spent on social activities declined when allowing access to the outdoor enclosure, at least in the beginning of the period, which could be due to an increased available space. The lower level of aggressive interactions when the goats had no access to the enclosure might be due to the completely barren environment inside with very few resources to compete for or because the high animal density inside make them less responsive. Furthermore, because the goats spent most of their active time in the enclosure, this area was clearly perceived as attractive. Combined with a limited and defendable amount of branches the area could be an important resource that was worth fighting for. When designing outdoor enclosures it will thus be important to consider access to and distribution of enrichment items in the area. It should be noticed that most of these aggressive interactions outside were considered mild and short, although this was not recorded systematically. Sometimes it was also difficult to distinguish between play-fighting and fighting with a more aggressive nature as the nature of the interactions seemed to be different from what is usually observed inside.

## Conclusions

In conclusion, the goats preferred to use the outside enclosure when being active, and branches were perceived as an attractive enrichment. Although the total time spent on social activities declined with access to the enclosure, aggression level increased compared to the barren and high-animal-density inside environment. This could be due to a higher motivation to defend attractive resources as the amount of branches was indeed limited and defendable or that the increased space allowance and the enriched environment increased play-fighting.

## Competing interests

The authors declare that they have no competing interests.

## Authors’ contributions

RE and ILA carried out the experiments. KEB and ILA planned the experimental design and participated in preparing the manuscript. KEB performed the data analysis and did the statistical analysis. All authors read and approved the final manuscript.
